# Transcriptomic changes in mouse embryonic stem cells exposed to thalidomide during spontaneous differentiation

**DOI:** 10.1016/j.dib.2015.05.014

**Published:** 2015-06-03

**Authors:** Xiugong Gao, Robert L. Sprando, Jeffrey J. Yourick

**Affiliations:** Division of Toxicology, Office of Applied Research and Safety Assessment, Center for Food Safety and Applied Nutrition, U.S. Food and Drug Administration, Laurel, MD, USA

**Keywords:** Thalidomide, Transcriptomics, Embryonic stem cell, Developmental toxicity, Differentiation, Microarray, Mouse

## Abstract

Thalidomide is a potent developmental toxicant that induces a range of birth defects, notably severe limb malformations. To unravel the molecular mechanisms underpinning the teratogenic effects of thalidomide, we used microarrays to study transcriptomic changes induced by thalidomide in an in vitro model based on the differentiation of mouse embryonic stem cells (mESCs), and published the major findings in a research article entitled “Thalidomide induced early gene expression perturbations indicative of human embryopathy in mouse embryonic stem cells” [1]. The data presented herein contains complementary information related to the aforementioned research article.

Specifications table

**Table:** 

Subject area	Biology
More specific subject area	Toxicogenomics
Type of data	1) Processed microarray data in .CEL format 2) Excel spreadsheet files listing identified genes, GO terms and functional clusters
How data was acquired	Microarray data generated on Affymetrix Mouse Gene 2.0 ST Array
Data format	Processed or analyzed
Experimental factors	Induction of spontaneous differentiation was achieved through embryoid body (EB) formation in hanging drop culture following a procedure adapted from De Smedt et al.[2]
Experimental features	Cells were collected at 24, 48, and 72 h after exposure to 0.25 mM thalidomide. Total RNA (50 ng) were preprocessed using the Affymetrix GeneChip WT PLUS Reagent Kit and hybridized onto the Affymetrix Mouse Gene 2.0 ST Array
Data source location	Laurel, MD, USA
Data accessibility	The analyzed data is with this article. Processed microarray data (.CEL files) can be accessed at Gene Expression Omnibus with accession number GSE61306 (http://www.ncbi.nlm.nih.gov/geo/query/acc.cgi?acc=GSE61306)

Value of the data

• The data represent the first toxicogenomic study on thalidomide using mouse embryonic stem cells [1].
• The gene expression data provide insights into mechanisms of thalidomide embryotoxicity [1].
• The functions and pathways associated with thalidomide-impacted genes conform to known thalidomide clinical outcomes [1].
• The data suggest that transcriptomics coupled with mouse embryonic stem cells is a valuable model for developmental toxicity testing [1].

## 1 Experimental design, materials and methods

### 1.1 Materials

(±)-Thalidomide ((RS)-2-(2,6-dioxopiperidin-3-yl)-1H-isoindole-1,3(2H)-dione) and all other chemicals used in this study were of molecular biology grade and were obtained from Sigma-Aldrich (St. Louis, MO) unless otherwise stated.

### 1.2 Pluripotent mouse embryonic stem cell culture

Pluripotent ESGRO Complete Adapted C57BL/6 mESCs, which have been pre-adapted to serum-free and feeder-free culture condition, were obtained from EMD Millipore (Billerica, MA) at passage 12 (with 80% normal male mouse karyotype). The cells were seeded in cell culture flasks (Nunc, Roskilde, Denmark) coated with 0.1% gelatin solution (EMD Millipore), and maintained at 37 °C in a 5% CO_2_ humidified incubator at standard densities (i.e., between 5×10^4^/cm^2^ and 5×10^5^/cm^2^) in ESGRO Complete Plus Clonal Grade Medium (EMD Millipore). The medium contains leukemia inhibitory factor (LIF), bone morphogenic protein 4 (BMP-4), and a glycogen synthase kinase-3b inhibitor (GSK3b-I) to help maintain pluripotency and self-renewal of the ESCs. Cells were passaged every 2–3 days (when reaching 60% confluence) with ESGRO Complete Accutase (EMD Millipore) at about 1:6 ratio. C57BL/6 mESCs maintain a stable karyotype under the above passaging condition. The cells used in the current study were at passage 18.

### 1.3 Mouse embryonic stem cell differentiation through embryoid body formation

Induction of differentiation was achieved through embryoid body (EB) formation via hanging drop culture following a procedure adapted from De Smedt et al. [2]. In brief, stem cells were thawed and a suspension was prepared at a concentration of 3.75×10^4^ cells/ml in ESGRO Complete Basal Medium (EMD Millipore), which does not contain LIP, BMP-4, or GSK3b-I. About 50 drops (each of 20 µl) of the cell suspension were placed onto the inner side of the lid of a 10-cm Petri dish filled with 5 ml phosphate buffered saline (PBS; EMD Millipore) and incubated at 37 °C and 5% CO_2_ in a humidified atmosphere. After 3 days, EBs formed in the hanging drops (*Ø*330–350 μm) were subsequently transferred into 6-cm bacteriological Petri dishes (Becton Dickinson Labware, Franklin Lakes, NJ) for thalidomide exposure.

### 1.4 Thalidomide exposure and RNA isolation

ESC differentiation cultures were exposed from the EB stage at day 3 onwards to 0.25 mM thalidomide or vehicle (0.25% DMSO) for 3 days. Preliminary results showed that DMSO at 0.25% (v/v) had no significant effect on gene expression during C57BL/6 ESC differentiation under the condition used in the study (data not shown).Thalidomide-exposed cultures and vehicle controls were collected at 24 h, 48 h, and 72 h (culture day 4, 5, and 6). Three biological replicates were used for each condition. Treatment with thalidomide did not affect EB sizes (data not shown). EBs were lysed in RLT buffer (Qiagen; Valencia, CA) supplemented with β-mercaptoethanol, homogenized by QIAshredder (Qiagen), and kept in a −80 °C freezer until further processing. Total RNA was isolated on the EZ1 Advanced XL (Qiagen) automated RNA purification instrument using the EZ1 RNA Cell Mini Kit (Qiagen) following the manufacturer׳s protocol, including an on-column DNase digestion. RNA concentration and purity (260/280 ratio) were measured with the NanoDrop 2000 UV–Vis spectrophotometer (NanoDrop Products, Wilmington, DE). Integrity of RNA samples was assessed by the Agilent 2100 Bioanalyzer (Santa Clara, CA) with the RNA 6000 Nano Reagent Kit from the same manufacturer.

### 1.5 RNA processing and microarray experiment

The total RNA samples were preprocessed for hybridization to Mouse Gene 2.0 ST Array (Affymetrix, Santa Clara, CA) using the GeneChip WT PLUS Reagent Kit (Affymetrix) following the manufacturer׳s protocol. In brief, 50 ng of total RNA was used to generate first strand cDNA using reverse transcriptase and primers containing a T7 promoter sequence. The single-stranded cDNA was then converted to double-stranded cDNA by using DNA polymerase and RNase H to simultaneously degrade the RNA and synthesize second-strand cDNA. Complimentary RNA (cRNA) was synthesized and amplified by in vitro transcription (IVT) of the second-stranded cDNA template using T7 RNA polymerase. Subsequently, sense-strand cDNA was synthesized by the reverse transcription of cRNA with incorporated deoxyuridine triphosphate (dUTP). Purified, sense-strand cDNA was fragmented by uracil-DNA glycosylase (UDG) and apurinic/apyrimidinic endonuclease 1 (APE 1) at the unnatural dUTP residues and labeled by terminal deoxynucleotidyl transferase (TdT) using the Affymetrix proprietary DNA Labeling Reagent that is covalently linked to biotin. Subsequent hybridization, wash, and staining were carried out using the Affymetrix GeneChip Hybridization, Wash, and Stain Kit and the manufacturer׳s protocols were followed. Briefly, each fragmented and labeled sense-strand cDNA target sample (approximately 3.5 µg) was individually hybridized to a GeneChip Mouse Gene 2.0 ST Array at 45 °C for 16 h in Affymetrix GeneChip Hybridization Oven 645. After hybridization, the array chips were stained and washed using an Affymetrix Fluidics Station 450. The chips were then scanned on Affymetrix GeneChip Scanner 3000 7G and the image (.DAT) files were preprocessed using the Affymetrix GeneChip Command Console (AGCC) software v.4.0 to generate cell intensity (.CEL) files. Prior to data analysis, all arrays referred to in this study were assessed for data quality using the Affymetrix Expression Console software v.1.3 and all quality assessment metrics (including spike-in controls during target preparation and hybridization) were found within boundaries. The data set has been deposited in Gene Expression Omnibus (GEO; http://www.ncbi.nlm.nih.gov/geo/) of the National Center for Biotechnology Information with accession number GSE61306.

### 1.6 Data processing and statistical analysis

The values of individual probes belonging to one probe set in .CEL files were summarized using the robust multi-array average (RMA) algorithm [3] embedded in the Expression Console software v.1.3 (Affymetrix), which comprises of convolution background correction, quantile normalization, and median polish summarization. Subsequently, differentially expressed genes (DEGs) were selected using one-way analysis of variance (ANOVA) using the Affymetrix Transcriptome Analysis Console (TAC) software v.1.0. The fold change (FC) of every gene, together with their corresponding *p*-Value, was used for selection of DEGs with cutoff values indicated in the text.

### 1.7 Gene ontology analysis

The significantly regulated genes were subjected to gene ontology (GO) using the Database for Annotation, Visualization, and Integrated Discovery (DAVID) [4], [5] to find overrepresentations of GO terms in the biological process (BP) category at all levels (GOTERM_BP_ALL) and associated clusters. As background, the *Mus musculus* (mouse) whole genome was used. Statistical enrichment was determined using default settings in DAVID.

## 2 Deposited data

The processed data (.CEL files) can be found here: http://www.ncbi.nlm.nih.gov/geo/query/acc.cgi?acc=GSE61306.

## Conflict of interest

The authors declare that there are no conflicts of interest.

## Appendix A Supplementary materials

Supplementary data Table 1. Full list of DEGs at different time points during ESC differentiation upon exposure to thalidomide. Differentiating EBs were treated with 0.25 mM thalidomide for 3 days, and global gene expression analysis was conducted at 24, 48, and 72 h in thalidomide-exposed samples relative to their time-matched controls. Using cut-off criteria of *p*<0.05 and |FC|>1.5, a total of 214, 230, and 56 DEGs were identified at the three time points respectively. The values in the columns under the titles of *Thalidomide* and *Control* are the Bi-weight average signals for the DEGs on log_2_ scale under each condition. A negative FC value means downregulation, whereas a positive value indicates upregulation.

Supplementary data Table 2. Lists of GO terms in the biological process (BP) category at all levels (GOTERM_BP_ALL) enriched from the downregulated DEGs affected by thalidomide at various time points during ESC differentiation. DAVID was used for the analysis using the downregulated DEGs identified during ESC differentiation (see text and Table S1 for details). The *Mus musculus* (mouse) whole genome was used as background. Statistical enrichment was determined through a modified Fisher׳s exact test (*p*<0.1) and count threshold>2 (default settings in DAVID). The column under *Count* indicates the number of DEGs involved in the specific GO term, while the column under % indicates the number of DEGs involved in the GO term as a percentage of the total number of downregulated DEGs at each time point. *Fold enrichment* means the number of genes (in the list) involved in a particular GO term as a percentage of total number genes of the list as compared to all genes involved in that particular GO term as a percentage of the whole genome (population background) [5].

Supplementary data Table 3. Lists of GO term clusters enriched by thalidomide downregulated DEGs at various time points during ESC differentiation. Functional Annotation Clustering in DAVID was used for the analysis with default settings. The *Mus musculus* (mouse) whole genome was used as background. See Supplementary Table 2 for the definition of terms.

Supplementary data Table 4. Lists of GO terms in the biological process (BP) category at all levels (GOTERM_BP_ALL) enriched from the upregulated DEGs affected by thalidomide at various time points during ESC differentiation. DAVID was used for the analysis using the downregulated DEGs identified during ESC differentiation (see text and Table S1 for details). The *Mus musculus* (mouse) whole genome was used as background. Statistical enrichment was determined through a modified Fisher׳s exact test (*p*<0.1) and count threshold>2. See Supplementary Table 2 for the definition of terms.

Supplementary data Table 5. Lists of GO terms forming clusters relating to sensory perception of smell in the neurological system process enriched by upregulated genes at 24 h and 48 h. Functional Annotation Clustering in DAVID was used for the analysis with default settings. The *Mus musculus* (mouse) whole genome was used as background. See Supplementary Table 2 for the definition of terms.
